# Disruption of Macrodomain Protein SCO6735 Increases Antibiotic Production in Streptomyces coelicolor*

**DOI:** 10.1074/jbc.M116.721894

**Published:** 2016-09-15

**Authors:** Jasna Lalić, Melanija Posavec Marjanović, Luca Palazzo, Dragutin Perina, Igor Sabljić, Roko Žaja, Thomas Colby, Bruna Pleše, Mirna Halasz, Gytis Jankevicius, Giselda Bucca, Marijan Ahel, Ivan Matić, Helena Ćetković, Marija Luić, Andreja Mikoč, Ivan Ahel

**Affiliations:** From the ‡ Division of Molecular Biology,; the ¶ Division of Physical Chemistry, and; the ‖ Division for Marine and Environmental Research, Ruđer Bošković Institute, Zagreb 10002, Croatia,; the § Sir William Dunn School of Pathology, University of Oxford, Oxford OX1 3RE, United Kingdom,; the ** Max Planck Institute for Biology of Ageing, D-50931 Cologne, Germany, and; the ‡‡ School of Pharmacy and Biomolecular Sciences, University of Brighton, Huxley Building, Moulsecoomb, Brighton BN2 4GJ, United Kingdom

**Keywords:** ADP-ribosylation, antibiotics, DNA damage, enzyme mechanism, microbiology, nicotinamide adenine dinucleotide (NAD), post-translational modification (PTM), ADP-ribose, PARP, Streptomyces

## Abstract

ADP-ribosylation is a post-translational modification that can alter the physical and chemical properties of target proteins and that controls many important cellular processes. Macrodomains are evolutionarily conserved structural domains that bind ADP-ribose derivatives and are found in proteins with diverse cellular functions. Some proteins from the macrodomain family can hydrolyze ADP-ribosylated substrates and therefore reverse this post-translational modification. Bacteria and *Streptomyces*, in particular, are known to utilize protein ADP-ribosylation, yet very little is known about their enzymes that synthesize and remove this modification. We have determined the crystal structure and characterized, both biochemically and functionally, the macrodomain protein SCO6735 from *Streptomyces coelicolor*. This protein is a member of an uncharacterized subfamily of macrodomain proteins. Its crystal structure revealed a highly conserved macrodomain fold. We showed that SCO6735 possesses the ability to hydrolyze PARP-dependent protein ADP-ribosylation. Furthermore, we showed that expression of this protein is induced upon DNA damage and that deletion of this protein in *S. coelicolor* increases antibiotic production. Our results provide the first insights into the molecular basis of its action and impact on *Streptomyces* metabolism.

## Introduction

Protein ADP-ribosylation is a reversible post-translational modification in which an ADP-ribose moiety from NAD^+^ is transferred to a target protein. The covalent attachment of one ADP-ribose leads to mono-ADP-ribosylation, whereas the transfer of additional ADP-ribose molecules through *O-*glycosidic ribose-ribose bonds results in the synthesis of poly-ADP-ribose (PAR)^4^ polymers. The families that generate protein ADP-ribosylation, poly-ADP-ribose polymerases (PARPs), mono-ADP-ribosyltransferases, and certain sirtuins, in eukaryotes regulate a variety of cellular processes, such as DNA repair, transcription, regulation of centromere function, telomere length and aging, protein degradation, apoptosis, and necrosis (1–5). Poly-ADP-ribose glycohydrolase (PARG) is one of the proteins capable of removing ADP-ribosylation. PARG specifically cleaves PAR chains at *O-*glycosidic ribose-ribose bonds releasing ADP-ribose monomers or PAR oligomers, but it is unable to cleave the chemical bond between the proximal ADP-ribose unit and the modified proteins (6). Recent studies have demonstrated that a family of macrodomain proteins (MacroD1, MacroD2, and TARG1) can revert terminal, protein-proximal glutamate-linked mono-ADP-ribosylation (1, 7–9). TARG1, MacroD1/2, and PARG represent different subgroups within the macrodomain protein family (10). Macrodomains are ancient folds with high affinity for ADP-ribose binding (11, 12). These three macrodomain subgroups use the macrodomain for catalysis but with distinct mechanisms (reviewed in Ref. 1). Furthermore, an evolutionarily distinct protein that can catalyze the removal of the protein-proximal arginine-linked mono-ADP-ribosylation is a mono-ADP-ribosylhydrolase (ARH) 1 (13). Importantly, reversal of post-translational modification has been established as important in many cellular processes (7, 14, 15).

Although ADP-ribosylating bacterial toxins, which act by irreversibly modifying crucial host cell proteins, were discovered over 40 years ago (reviewed in Ref. 16), reversible mono-ADP-ribosylation and its role in signaling in bacteria is generally not well understood. Physiologically important, non-toxic mono-ADP-ribosylation has been extensively studied only in nitrogen-fixing bacteria *Rhodospirillum rubrum*, where the enzymes dinitrogenase reductase mono-ADP-ribose transferase and dinitrogenase reductase-activating glycohydrolase (DraG) regulate nitrogen fixation depending on nitrogen availability and energy status of the cell (17). Being the best characterized, bacterial DraG (homologue of human ARH1 and ARH3) is a representative of the group of arginine-specific ADP-ribosylhydrolases whose homologues are distributed across all three domains of life. Endogenous ADP-ribosylation has also been reported for some other bacteria, *Myxococcus xanthus* (18, 19), *Mycobacterium smegmatis* (20), *Bacillus subtilis* (21), and *Streptomyces* representatives (22–25), but little is known about its function in bacteria. Genomic evidence indicates that proteins involved in ADP-ribosylation processing are widespread among bacteria. Although PARP homologues are found rarely, PARG and other macrodomain protein homologues are found more often, suggesting that protein ADP-ribosylation is more common than previously thought (10). So far, the most evidence for intracellular endogenous protein ADP-ribosylation has been found in *Streptomyces* species. *Streptomyces* are soil bacteria well known for their complex life cycle, which includes morphological differentiation and production of secondary metabolites including antibiotics, anti-cancer drugs and immunosuppressors. In *Streptomyces griseus* and *Streptomyces coelicolor*, just like in other bacteria in which ADP-ribosylation has been studied, ADP-ribosylation patterns change with morphological differentiation and are related to changes in metabolic requirements (24, 26). However, almost nothing is known about transferases and hydrolases responsible for the reversible ADP-ribosylation in these organisms.

Thus far, no proteins have been identified that have the ability to reverse (hydrolyze) protein ADP-ribosylation in *Streptomyces* and most other bacterial organisms. Here, we analyzed the *S. coelicolor* genome and found a number of possible candidates, including homologues of human PARG and MacroD1, as well as an uncharacterized type of macrodomain protein, SCO6735. We have focused our research on SCO6735 because we could show that it encompasses a macrodomain evolutionarily close to the ALC1 (amplified in liver cancer)/TARG1 (terminal ADP-ribose protein glycohydrolase) macrodomain group. Here we determine the structure of SCO6735 and show that this protein hydrolyzes glutamate-linked protein mono-ADP-ribosylation. Furthermore, we show that depletion of SCO6735 leads to a significantly increased production of antibiotic actinorhodin in *S. coelicolor*.

## Results

### 

#### 

##### Putative ART and ARHs in Streptomyces

Our genomic analyses and homology searches predict the existence of only one potential ART in *S. coelicolor*, SCO5461, which confirms previous findings (27). SCO5461 is a highly diverged PARP homologue most similar to pierisins and mosquitocidal toxin and can modify guanosine and guanine mononucleotides *in vitro*, whereas its ability to directly modify proteins and *in vivo* substrates in general has not been clarified (28). SCO5461 is a secreted protein and is not found in *S. griseus* and most other *Streptomyces* species; thus it cannot be the major cellular protein ART in streptomycetes.

On the other hand, our analyses revealed a number of putative ADP-ribosyl protein hydrolases in *S. coelicolor* and other streptomycetes, suggesting a lively ADP-ribosylation metabolism. We identified both DraG-like (SCO0086, SCO1766, SCO2028, SCO2029, SCO2030, SCO4435, and SCO5809) and macrodomain containing (SCO0909, SCO6450, and SCO6735) hydrolases. Two of three macrodomain proteins have predicted functions. The SCO0909 protein of *S. coelicolor* is a bacterial-type PARG, predicted to remove poly-ADP-ribosylation (6); whereas SCO6450 is a macrodomain protein predicted to remove mono-ADP-ribosylation, it is a homologue of the MacroD proteins, which are present in representatives from all three domains of life (10, 12). We focused our research on the third macrodomain protein, SCO6735. SCO6735 is a 161-amino acid protein with molecular weight of 17.4 kDa that belongs to an uncharacterized subgroup of macrodomain proteins. The orthologues of SCO6735 are less widely distributed than MacroD orthologues but still found in several bacterial phyla (Bacteroidetes, Firmicutes, and Planctomycetes). They are most frequent among Actinobacteria.

##### SCO6735 Is a Macrodomain Protein That Groups into ALC1/TARG1 Branch

**FIGURE 1.**
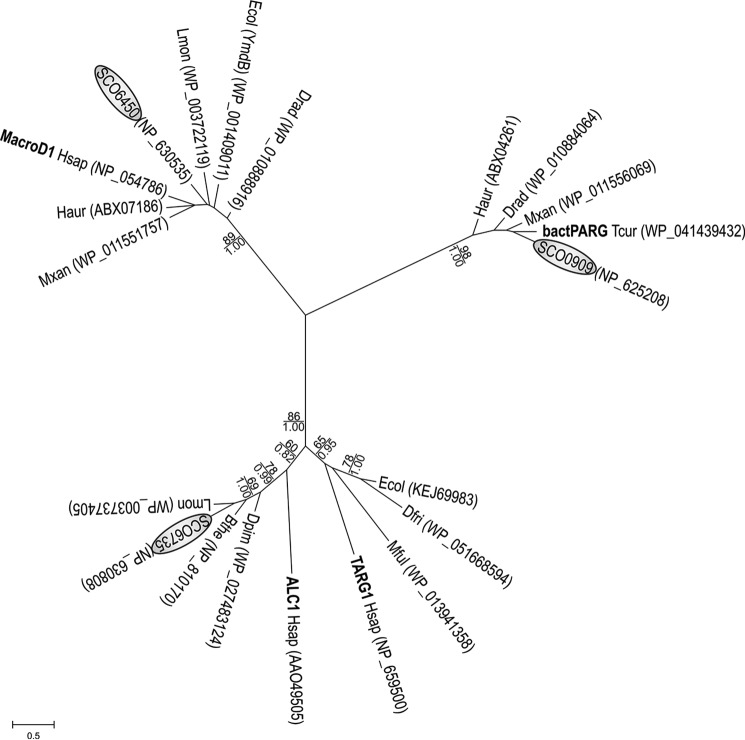
**Maximum likelihood phylogenetic tree illustrating the relationship between selected representative macrodomains.** Human proteins (*Hsap*) and bacterial PARG from *Thermomonospora curvata* (*Tcur*) are shown in *bold type. S. coelicolor* proteins are *circled*. Remaining macrodomain proteins were selected from the following bacterial species: *B. thetaiotaomicron* (*Bthe*), *Deinococcus frigens* (*Dfri*), *Deinococcus pimensis* (*Dpim*), *D. radiodurans* (*Drad*), *E. coli* (*Ecol*), *Herpetosiphon aurantiacus* (*Haur*), *Listeria monocytogenes* (*Lmon*), *Myxococcus fulvus* (*Mful*), and *M. xanthus* (*Mxan*). Sequence accession numbers are given in *parentheses*. Bootstrap values ML (>60%) are given above the *lines*, and MCMC values are given (>0.6) below the *lines*. The *scale bar* indicates the genetic distance of the branch lengths.

We performed phylogenetic analysis using selected bacterial macrodomain proteins and human representatives of different macrodomain groups. Phylogenetic analyses showed that the SCO6735 groups into the ALC1/TARG1 branch (Fig. 1). ALC1 is a chromatin remodeler that uses its macrodomain to bind DNA damage induced poly-ADP-ribosylation (29). TARG1 removes PARP-dependent mono-ADP-ribosylation (7). SCO6735 shares 27% identity and 45% similarity with the human ALC1 macrodomain and 13% identity and 28% similarity with the TARG1 macrodomain.

**FIGURE 2.**
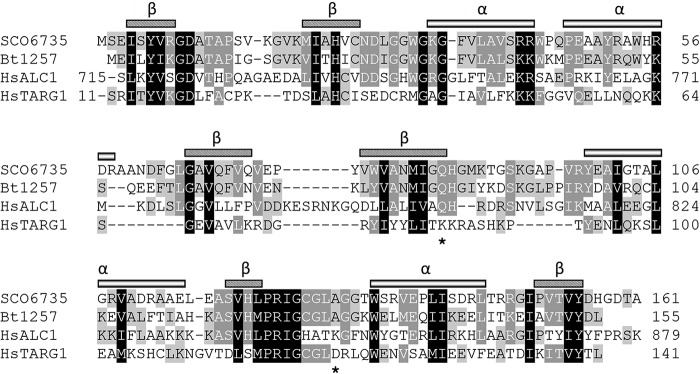
**Structure-based alignment of selected macrodomain protein sequences.** Designated secondary structure elements refer to SCO6735. Residues responsible for binding and hydrolysis of ADP-ribose in human TARG1 (Lys^84^ and Asp^125^) are marked with *asterisks*.

Sequence alignment (Fig. 2) revealed conserved residues shared by SCO6735 and protein BT1257, from *Bacteroides thetaiotaomicron*, the most similar bacterial protein with a known crystal structure (unpublished, PDB code 2FG1). These two proteins share 48% identity and 62% similarity, and we assumed that the hypothetical protein BT1257 is likely the SCO6735 orthologue. It is worth noting that the residues important for ADP-ribose binding (Lys^84^) and hydrolytic activity (Asp^125^) of human TARG1 are not conserved in SCO6735 and BT1257 proteins.

##### Biochemical Activity of SCO6735 Protein

**FIGURE 3.**
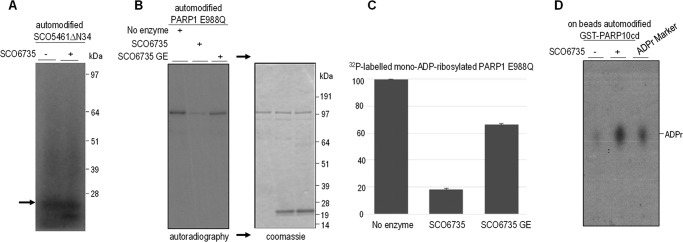
**Activity of SCO6735 protein.** *A*, activity of SCO6735 on ^32^P-automodified SCO5461ΔN34 substrate. The *arrow* indicates the position of SCO5461ΔN34 protein. *B*, activity of SCO6735 on automodified PARP1 E988Q protein. Recombinant PARP1 E988Q (0.5 μm) was automodified in presence of [^32^P]NAD^+^ and treated or not with 1 μm of SCO6735 or with 1 μm of SCO6735 G128E mutant. *Right panel*, Coomassie staining of dried gel exposed in *left panel. C*, quantification of residual ^32^P-labeled ADP-ribose on PARP1 E988Q after treatment with buffer, SCO6735 or SCO6735 G128E mutant. The ratio between the intensity of each autoradiography band and correspondent intensity in Coomassie staining data are the means ± S.D. of the values obtained in two independent experiments. *D*, TLC analyzing the by-products of incubation of GST-PARP10cd immobilized on beads with buffer or SCO6735. ADP-ribose marker was obtained treating recombinant wild type PARP1 with recombinant human PARG enzyme.

We wanted to assess whether SCO6735 has the ability to hydrolyze protein ADP-ribosylation, as has been demonstrated for some other macrodomain family members. First, we sought to determine whether the only predicted ART in *S. coelicolor*, SCO5461, can modify itself *in vitro*. Using an ADP-ribosyltransferase assay with [^32^P]NAD^+^ as an ADP-ribose donor and the purified SCO5461ΔN34, we showed significant automodification activity. However, mono-ADP-ribosylated SCO5461ΔN34 was not a substrate for SCO6735, because we observed no decrease of the radioactive signal (Fig. 3*A*). We performed mass spectrometry analysis and identified aspartate 161 as a modification site on the auto-ADP-ribosylated SCO5461ΔN34. Next, we used a (heterologous) model substrate: mono-ADP-ribosylated PARP1 E988Q mutant, which is known to be modified mostly on glutamate residues (7). Using this substrate, we observed removal of radioactive signal suggesting enzymatic activity of SCO6735 against mono-ADP-ribosylated proteins modified on glutamate residues (Fig. 3*B*). SCO6735 hydrolytic activity was partially abolished by a mutation of glycine 128 in glutamate (Fig. 3, *B* and *C*). To analyze the nucleotide product of SCO6735 activity, we used the automodified GST-PARP10cd as a substrate and assayed the reaction products by thin layer chromatography (Fig. 3*D*). Our data showed that a major by-product of hydrolytic reaction is ADP-ribose, as seen for other macrodomain hydrolases (12). However, because the catalytic residues found in other macrodomain proteins such as TARG1 are not conserved in SCO6735 (Fig. 2), we conclude that the catalytic mechanism should be unique for the SCO6735 macrodomain subclass.

##### Structure of SCO6735 Confirms Different Catalytic Residues Compared with TARG1

**FIGURE 4.**
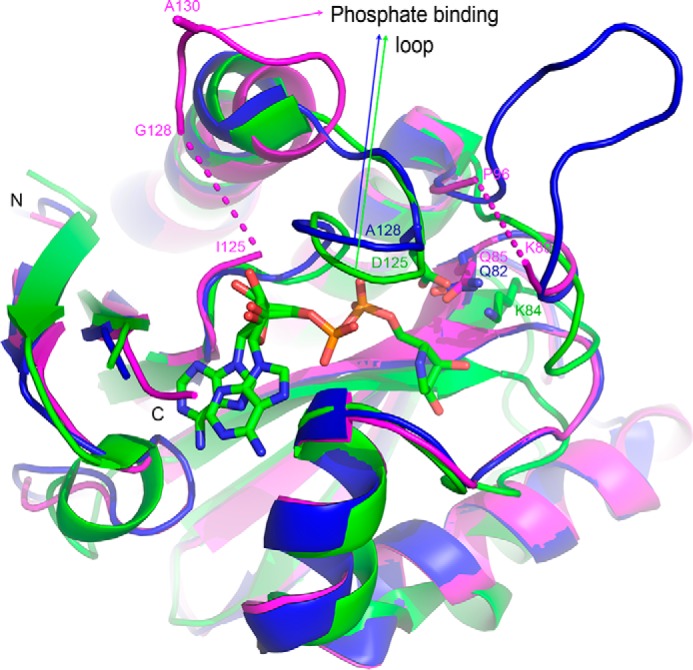
**Three-dimensional structure of SCO6735 (*magenta*) superimposed on a human TARG1/C6orf130 (PDB code 4J5R, *green*) and bacterial, *B. thetaiotaomicron* (PDB code 2FG1, *blue*) proteins.** Human protein is in complex with PARG inhibitor ADP-HPD. ADP-HPD, as well as amino acids important for ligand binding (Lys^84^) and catalytic activity (Asp^125^) and corresponding amino acids in bacterial structures (Gln^85^ and Ala^130^ in SCO6735 and Gln^82^ and Ala^128^ in BT1257) are shown in *stick representation*. Amino acids that could not be well defined in the electron density maps are omitted and shown by *dashed line*.

To better understand the structure-function relationship of SCO6735 hydrolytic activity, we determined a high resolution (1.60 Å) crystal structure of this protein (Fig. 4). The structure of SCO6735 revealed highly conserved three-layered α-β-α sandwich macrodomain fold with a deep cleft that, by analogy with other macrodomain proteins, represents a putative ligand-binding site. A central six-stranded β-sheet contains a mixture of five parallel and one anti-parallel strand, and it is surrounded by four α-helices. Structure alignment using PDBeFold (30) reveals many structural homologues within the PDB. Those most similar to SCO6735 are the structure of protein BT1257 from *B. thetaiotaomicron* (PDB code 2FG1; *Z* score of 14.4; RMSD 0.77) and the structure of human TARG1/C6orf130 (PDB code 4J5R; *Z* score of 9.4; RMSD 2.04) (7). Structural alignment of these three structures shows a high degree of similarity among them, with significant differences only in the loop regions.

The most pronounced difference is observed in the phosphate binding loop position. It was previously shown that, as for example in TARG1/C6orf130 protein, in both apo and complexed structures, the phosphate binding loop has the same position and encloses substrate within the active site (7). Surprisingly, in SCO6735 this loop is located far away (∼15 Å) from the binding cleft (Fig. 4). Two amino acids from this loop, Gly^126^ and Cys^127^, which were not well defined in the electron density maps, are omitted. B-factors in phosphate binding loop region are not significantly higher than B-factors of the surrounding amino acids, as also observed for TARG1/C6orf130 and BT1257 apo proteins, indicating similar flexibility of this loop in all three considered structures.

For TARG1/C6orf130 protein also NMR solution structures for both apo and ADP-ribose-bound complex are available in the literature (31). Interestingly, in none of the 20 final NMR conformers from apo and 20 from the complexed protein phosphate binding loop reaches a position so far away from the binding cleft as in SCO6735 structure. To check that the position of the phosphate binding loop in our structure is not a consequence of crystal packing, we carefully checked all contacts between amino acids composing the phosphate binding loop and surrounding symmetry equivalent molecules. We found out that there is only one hydrogen bond (Leu^129^ N-H … O^δ1B^ Asp^156^). There are no polar amino acids in the phosphate binding loop (^123^RIGCGLAGG^133^), except for the first arginine residue, suggesting that it is not very likely that electrostatic contacts are responsible for phosphate binding loop configuration. The same is valid for hydrophobic interactions caused by a lack of hydrophobic residues in this loop.

A structural comparison shows that the catalytic residues of TARG1 (Lys^84^ and Asp^125^) are not conserved in SCO6735 and BT1257 (replaced by Gln and Ala, respectively), additionally confirming that these proteins utilize different catalytic mechanism. Up to now we were not able to obtain the SCO6735 structure with bound ADP-ribose.

##### SCO6735 Is UV-inducible

**FIGURE 5.**
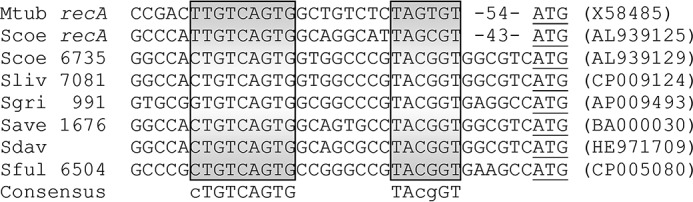
**Alignment of the RecA-NDp promoter sequences of *Mycobacterium tuberculosis* (*Mtub*) and *S. coelicolor* (*Scoe*) *recA* genes with SCO6735 and its orthologues in other *Streptomyces* species.** *Sliv*, *S. lividans*; *Sgri*, *S. griseus*; Save, *Streptomyces avermitilis*; *Sdav*, *Streptomyces davawensis*; *Sful*, *Streptomyces fulvissimus*. Putative promoter sequences are *boxed*, and the (potential) transcription start sites are *underlined*. The *numbers* indicate the distance to the starts of translation with respect to the shown sequences. GenBank^TM^ accession numbers for the sequences are given in *parentheses*.

To further understand the physiological function of SCO6735, we analyzed its promoter region to get an insight into the transcriptional regulation of the gene product. Strikingly, in the intergenic region, upstream of the SCO6735 gene, we found a highly conserved RecA-NDp type of promoter. Because this type of promoter precedes numerous genes involved in the DNA damage response in *Actinomycetales* (32, 33), it strongly suggested connection of SCO6735 macrodomain protein with DNA damage response in *Streptomyces*. Furthermore, the RecA-NDp promoter is also found upstream of SCO6735 homologues in several other *Streptomyces* species (Fig. 5).

**FIGURE 6.**
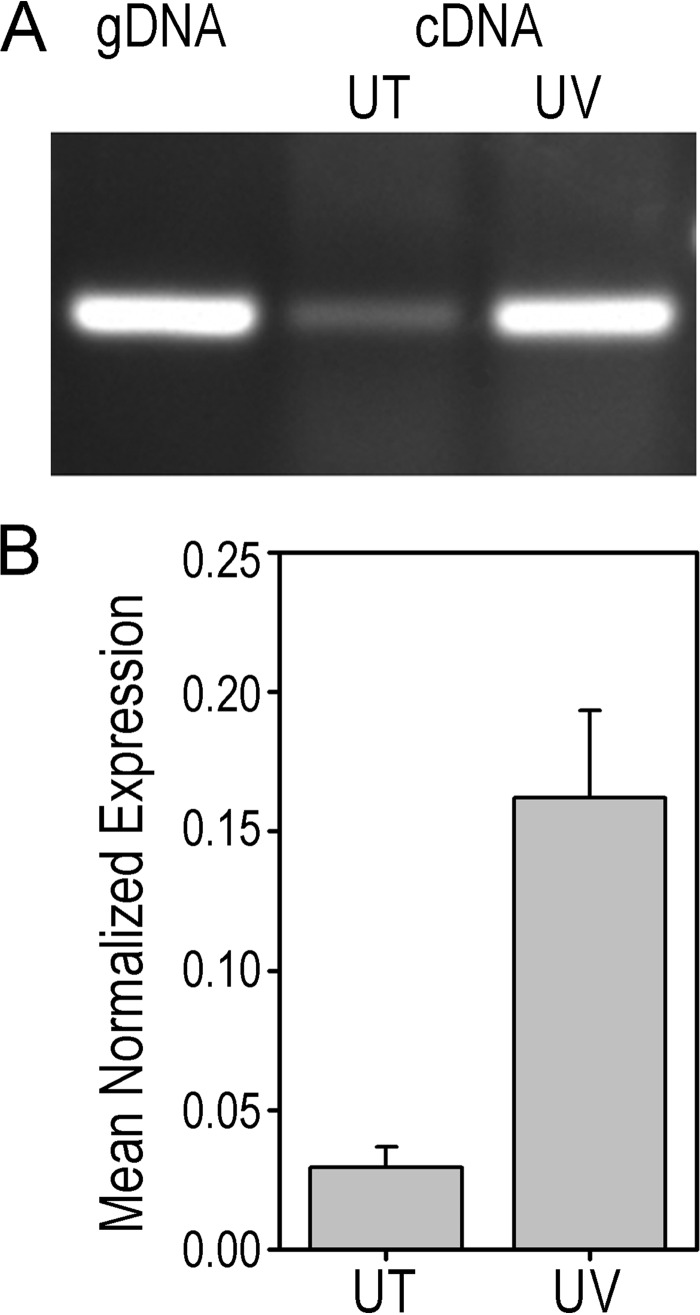
**Expression of SCO6735 gene is up-regulated upon DNA damage.** cDNA from untreated (*UT*) and UV-irradiated (200 J m^−2^) mycelium (*UV*) of *S. coelicolor* wild type strain was used as a template for PCR (*A*) and qRT-PCR analyses (*B*). *S. coelicolor* genomic DNA (*gDNA*) was used as a control template in PCR analysis. The data represent the mean values from three independent experiments. The *error bars* represent standard error of the mean.

We tested whether the SCO6735 gene is inducible by the SOS response. Regulation of SCO6735 gene expression upon DNA damage was analyzed using UV light as a DNA-damaging agent. Using a PCR analysis (Fig. 6*A*), we observed that expression of the SCO6735 gene in the *S. coelicolor* WT strain was notably increased after UV irradiation. To quantify this observation, we performed quantitative real time PCR analysis using a gene for 16S rRNA as an invariant endogenous control and *recA* gene as a positive control. Quantitative real time PCR (Fig. 6*B*) confirmed that the expression of the SCO6735 gene was significantly (5-fold) increased (two-tailed *t* test, *p* = 0.0337) after DNA damage caused by UV irradiation, thus implicating involvement of the SCO6735 protein in the DNA damage response.

##### Disruption of the SCO6735 Gene and Mutant Phenotype

The DNA damage response promoter suggested that SCO6735 protein may have a DNA repair function, so we decided to inactivate the gene encoding SCO6735 protein in *S. coelicolor* and analyze the phenotypes. We used the REDIRECT gene replacement procedure (34) to replace the SCO6735 gene from the *S. coelicolor* WT strain by an apramycin resistance cassette as described under “Experimental Procedures.” Disruption of the SCO6735 gene was confirmed by PCR, Southern hybridization of genomic DNA and qRT-PCR (data not shown). The deletion mutant used for further analysis was named *S. coelicolor* Δ6735.

**FIGURE 7.**
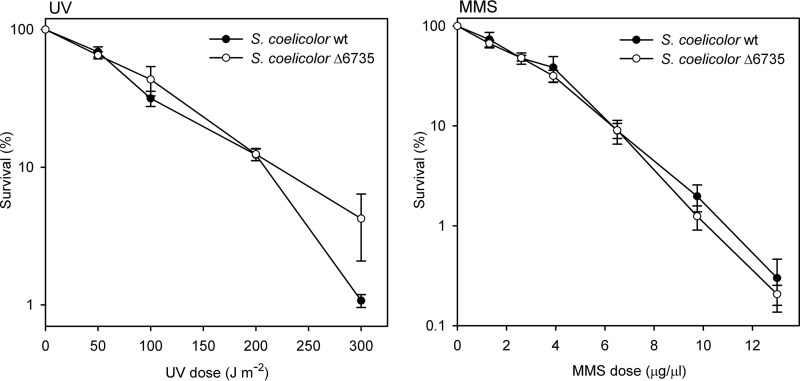
**UV and MMS sensitivity of the *S. coelicolor* Δ6735 mutant compared with wild type.** The data represent the mean values from three independent experiments. The *error bars* represent standard error of the mean.

To analyze whether the *S. coelicolor* Δ6735 strain exhibits sensitivity to DNA damage, we performed assays with two different mutagens. Spores of *S. coelicolor* WT and SCO6735 deletion mutant were irradiated with UV light (up to 300 J m^−2^) and treated with methyl methanesulfonate (MMS; up to 13 μg/μl) as described. Survival rates were determined as shown in Fig. 7. We did not notice significant differences in the survival rate between *S. coelicolor* WT and *S. coelicolor* Δ6735 strains after UV or MMS treatment. The absence of a DNA damage-sensitive phenotype could be a consequence of redundant pathways that can efficiently repair damage produced by UV-light and MMS. Alternatively, it is quite possible that SCO6735 is not directly involved in DNA repair.

**FIGURE 8.**
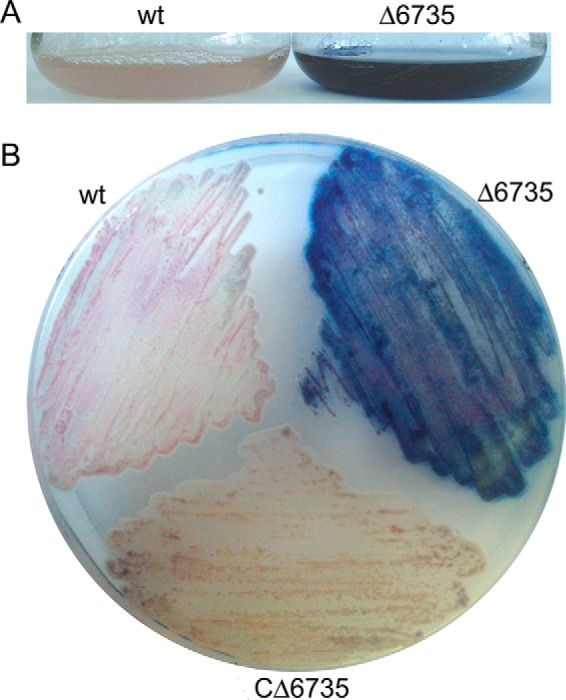
**Blue phenotype of *S. coelicolor* Δ6735 mutant in liquid (*A*) and on solid (*B*) minimal medium compared with wild type and complementation strain (CΔ6735) phenotypes.**

To explore other phenotypes, we assessed the growth of *S. coelicolor* Δ6735 on various culture media that might reveal changes in secondary metabolism. When grown on minimal medium, *S. coelicolor* Δ6735 showed a “blue phenotype,” suggesting accelerated and higher production of antibiotic actinorhodin when compared with the wild type strain (Fig. 8). To confirm that this phenotype is specifically due to disruption of SCO6735 function, we performed complementation analysis using ectopically expressed SCO6735. The SCO6735 gene, together with its RecA-NDp promoter region, was cloned into the site-specific integrating vector pMS82 and integrated into the *S. coelicolor* Δ6735 strain genome. The complementation strain, named *S. coelicolor* CΔ6735, showed reversion of the blue phenotype, indicating that the observed phenotype is a consequence of SCO6735 gene inactivation (Fig. 8).

##### Quantification of Actinorhodin Production in S. coelicolor Δ6735 Mutant

**FIGURE 9.**
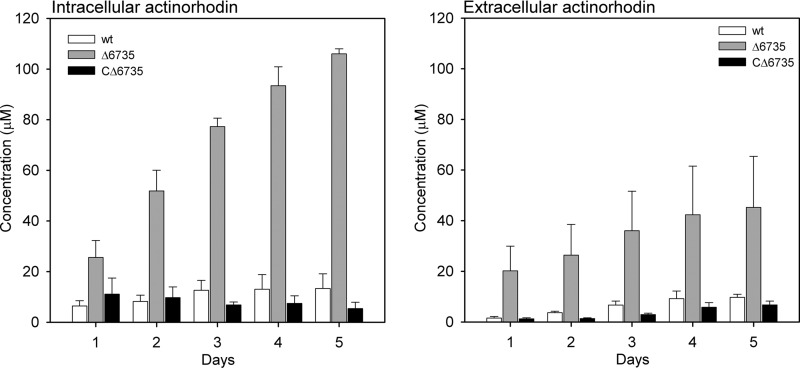
**The content of intracellular and extracellular actinorhodin in *S. coelicolor* WT, Δ6735 mutant, and complementation strain (CΔ6735) over 5 days of growth in minimal medium.** The data represent the mean values from three independent experiments. The *error bars* represent standard error of the mean.

Because the SCO6735-deficient strain showed a conditional effect on the production of antibiotic actinorhodin, we quantified the level of actinorhodin in *S. coelicolor* WT, SCO6735-deficient, and complementation strains. All strains were grown in liquid minimal medium for 5 days, and aliquots taken every 24 h were used to quantify intracellular and extracellular actinorhodin content. Pooled data for all days and measurements showed (Fig. 9) that actinorhodin levels in SCO6735-deficient mutant increased over time and were significantly higher compared with both the WT and complementation strains. *S. coelicolor* Δ6735 produced significantly more intracellular actinorhodin than both of the reference genotypes, on average 6.5 times more than the wild type and 8.72 times more than complementation strain (one-way ANOVA, *p* < 0.0001). Similar results were observed for the extracellular actinorhodin. The *S. coelicolor* Δ6735 strain produced on average 5.57 and 10.3 times more extracellular actinorhodin than the wild type and complementation strain, respectively (one-way ANOVA, *p* < 0.0001). Wild type and the complementation strain did not significantly differ in the levels of either intracellular (one-way ANOVA, *p* = 0.9232) or extracellular (one-way ANOVA, *p* = 0.8636) actinorhodin.

##### Deregulation of Actinorhodin-related Gene Expression in S. coelicolor Δ6735 Mutant

**FIGURE 10.**
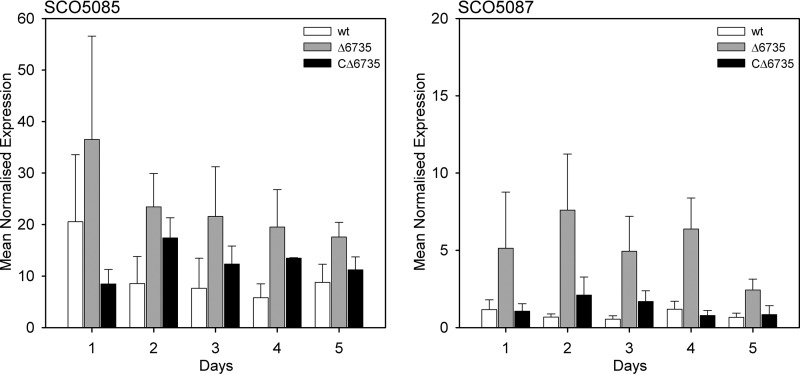
**Quantitative real time PCR analysis of genes involved in the biosynthesis of actinorhodin, SCO5085, and SCO5087 in *S. coelicolor* Δ6735 mutant, wild type and complementation strain.** Samples for RNA isolation were taken during the 5 days of growth in minimal medium. The data represent the mean values from three independent experiments. The *error bars* represent standard error of the mean.

Using qRT-PCR, we analyzed whether deficiency in SCO6735 protein influences expression of genes responsible for actinorhodin biosynthesis and regulation. Biosynthesis of the polyketide antibiotic actinorhodin is determined by ∼20 genes organized in five transcription units within the *act* gene cluster (35) dependent on the ActII-ORF4 protein, which binds to sequences in the target promoters (36). Two genes from the actinorhodin biosynthesis cluster that we included in our analysis were actinorhodin cluster activator protein (actII-ORF4) gene SCO5085 and α-subunit of actinorhodin polyketide β-ketoacyl synthase (actI-ORF1) gene SCO5087. Expression of these genes was analyzed in the *S. coelicolor* WT strain, SCO6735-deficient mutant, and the complementation strain. The strains were grown in liquid minimal medium for 5 days, and total RNA was isolated from the samples taken every 24 h. Our results (Fig. 10) confirmed that expression of both genes involved in actinorhodin biosynthesis was significantly increased in SCO6735-deficient strain compared with WT strain (two-tailed *t*-tests, *p* > 0.0148 and *p* > 0.0001 for SCO5085 and SCO5087, respectively). Wild type and the complementation strain did not significantly differ in the levels of both SCO5085 and SCO5087 gene expression (two-tailed *t*-tests, *p* = 0.6974 and *p* > 0.6579, respectively).

## Discussion

Very little is known about bacterial reversible ADP-ribosylation, with the exception of the role in nitrogen fixation in *Rhodospirillum* and *Azotobacter* species (17). Scattered evidence suggests that ADP-ribosylation in bacteria is much more common in regulating important cellular pathways, *e.g.* development and possibly mediating cell-cell contact in *M. xanthus* (18, 19), differentiation process in *B. subtilis* (21), and differentiation and secondary metabolism in *Streptomyces* (22, 37). Finally, here we suggest the possible involvement of the de-ADP-ribosylation process in the control of *S. coelicolor* antibiotic production. However, a direct role of SCO6735 protein and de-ADP-ribosylation reactions in this process remains to be experimentally verified. Transferases and hydrolases involved in ADP-ribosylation processes remain largely uncharacterized in bacteria. Genomic evidence indicates that only a small number of bacterial species possess all genes encoding proteins essential for a functional PAR metabolism, although there is no evidence that functional PAR metabolism really exists in bacteria. These genes were possibly acquired through horizontal gene transfer (6, 38), and bacteria might have evolved to use PARG as counteracting factor of the potentially harmful ADP-ribose/PAR present in the environment. PARGs are present in a scattering of bacteria. In *Deinococcus radiodurans* PARG is one of the highly induced proteins upon DNA damage caused by UV light (39). Protein mono-ADP-ribosylation is predictably more common in bacteria (10), where homologues of MacroD proteins are the most abundant representatives. Macrodomain protein YmdB from *Escherichia coli* seems to be a multifunctional protein that regulates RNase III activity and modulates bacterial biofilm formation (40).

Current genomic data suggest that ADP-ribosylation should be prominent in *Streptomyces*, with one possible ART and many ARHs. Even though ADP-ribosylation has been noted in *Streptomyces* more than 20 years ago, there is still very little mechanistic understanding of how ADP-ribosylation is regulated in these organisms. So far, only one ADP-ribosyltransferase has been identified in *Streptomyces*: SCO5461 protein. In accordance with structural similarity with two toxic ADP-ribosyltransferases, pierisins and mosquitocidal toxin, SCO5461 has been described as a DNA-modifying exotoxin, and its ADP-ribosylating activity toward N2 amino groups of guanine nucleosides, as well as mononucleotides, has been shown (28). Szirák *et al.* (37) have shown that disruption of SCO5461 leads to conditional pleiotropic phenotype characterized by morphological differentiation and antibiotic production defects. In addition, the same authors showed that deficiency in SCO5461 leads to a minor effect in protein ADP-ribosylation patterns; however, the question remained whether the effect was direct (37). Here we confirmed auto mono-ADP-ribosylating activity of the SCO5461 protein *in vitro*, but its specific protein trans targets are yet to be discovered. Several identified ADP-ribosylated proteins in *S. coelicolor* suggested a connection between protein ADP-ribosylation and the regulation of metabolic requirements of the cells (25, 41). SCO5461 is not conserved across the *Streptomyces* species (including *S. griseus*), suggesting that the major protein ADP-ribosyltransferases in *Streptomyces* are yet to be uncovered.

Surprisingly, there is evidence of a much larger number of potential ADP-ribose protein hydrolases in *S. coelicolor*. Seven of them are uncharacterized DraG homologues (SCO0086, SCO1766, SCO2028, SCO2029, SCO2030, SCO4435, and SCO5809), whereas three are macrodomain proteins (SCO0909, SCO6450, and SCO6735), representing three different groups of macrodomain superfamily. Although in eukaryotes macrodomains exist as compositions with other domains forming functionally complex proteins like ALC1, PARP9, and macroH2A1 (29, 42, 43), macrodomain proteins in bacteria are usually stand-alone proteins. Our preliminary results indicate that SCO6450, just like its human homologue MacroD1, indeed possesses enzymatic activity capable of removing ADP-ribose from mono-ADP-ribosylated protein. The SCO0909 protein is a bacterial-type PARG. Bacterial-type PARGs are much shorter than the proteins in human and higher eukaryotes and encompass only the PARG catalytic domain but are highly active and specific in hydrolyzing PAR *in vitro* (6). Our data showed that SCO6735 removes ADP-ribosylation from glutamate residues but does not reverse ADP-ribosylated SCO5461. This is further evidence that the relevant transferase(s) in *Streptomyces* are yet to be discovered. SCO6735 and its orthologue BT1257 represent a new subgroup of macrodomain proteins with a catalytic mechanism that is different from any known macrodomain proteins.

Our results, in accordance with previous findings, suggest the potential role of ADP-ribosylation in the regulation of metabolism (antibiotic production) in *Streptomyces*, whereas for the first time we have shown the possible involvement of ADP-ribosylation in response to DNA damage in *Streptomyces*. Involvement of ADP-ribosylation in the repair of DNA damage is well known and has been demonstrated in detail in eukaryotes (44, 45). A few lines of evidence suggest that ADP-ribosylation could also be involved in the DNA damage response in prokaryotes. In *M. smegmatis*, ADP-ribosyltransferase Arr was up-regulated in response to various stresses, including DNA damage (46). In *D. radiodurans*, PARG was up-regulated upon UV-induced DNA damage, pointing out that degradation of PAR could be more ancient than was initially considered (39). Gene SCO6735 is under control of the RecANDp promoter, and its expression is also up-regulated upon UV-induced DNA damage. Nevertheless, using two different mutagens (UV light and MMS), we were not able to show significant DNA damage-sensitive phenotype of the SCO6735-deficient mutant. This may not be unexpected given that *Streptomyces* possess multiple DNA repair systems that transcend bacterial systems and are closer to those of lower eukaryotes (47, 48).

To conclude, we believe our findings will help to understand ADP-ribosylation processes in *Streptomyces* (and bacteria in general) and establish *Streptomyces* as an important model to study bacterial ADP-ribosylation. Our data confirm that ADP-ribosylation in *Streptomyces* could be even more widely utilized than initially thought. For example, we uncovered possible involvement of de-ADP-ribosylation process in the control of antibiotic production and demonstrated a potential link between ADP-ribosylation and the DNA damage response.

## Experimental Procedures

### 

#### 

##### Bacterial Strains, Culture Conditions, and Plasmids

*S. coelicolor* M145 strain, a derivative of the wild type strain A3(2) lacking plasmids SCP1 and SCP2, was a generous gift from Prof. M. Bibb and was used in this study as a wild type strain (49). *S. coelicolor* strains were grown at 30 °C in liquid medium: complete regeneration medium (50) and minimal medium (MM) (49), and on solid media: mannitol soya flour, tryptic soy broth (TSB) (Difco), nutrient agar (Difco), and MM. For actinorhodin quantification and actinorhodin-related gene expression quantification, spores of different *S. coelicolor* strains were inoculated into complete regeneration medium and grown for 24 h. The next day, mycelia were washed in MM and continued incubation in 50 ml of MM for 5 days at 30 °C and 250 rpm. Every 24 h, aliquots were taken for the further analyses.

*E. coli* XL1-Blue (New England Biolabs) was used for all genetic manipulations. *E. coli* BL21(DE3) (Stratagene) was used for gene overexpression. Strains *E. coli* BW25113/pIJ790 and ET12567/pUZ8002 and cosmid St5F2A (51) used for gene disruption by REDIRECT technology (34) were obtained from the John Innes Centre (Norwich, UK). *E. coli* strains XL1-Blue and BL21(DE3) were grown in LB liquid and solid medium at 37 °C, whereas strain BW25113/pIJ790 at 30 °C until the loss of the plasmid with temperature sensitive origin of replication was needed. When necessary, the media were supplemented with antibiotics to the following concentrations: 100 μg/ml of ampicillin, 50 μg/ml of apramycin, 25 μg/ml of chloramphenicol, 50 μg/ml of kanamycin, 25 μg/ml of nalidixic acid, or 50 μg/ml of hygromycin. All antibiotics were purchased from Sigma-Aldrich. Plasmid pET15b (Novagen) was used for gene overexpression and *Streptomyces*-specific integrative vector pMS82 (52) for complementation analysis (a generous gift from Prof. M. Smith).

##### Gene Cloning, Mutagenesis, Overexpression, and Protein Purification

*S. coelicolor* genomic DNA was isolated as described (49) and used for PCR amplification of the gene encoding SCO6735 protein (NCBI, gene identifier 1102174) with primers 6735F (CGGTGGCCATATGTCGGAGATCAGCTATGTCC) and 6735R (GAGCCGCGGATCCCCTAGGCGGTGTCCCCG) that contain NdeI and BamHI restriction sites. PCR product was digested with the same restriction enzymes and cloned into pET15b. Specific point mutation was introduced using this plasmid construct, mutant primers 6735GEF (CCGCATAGGCTGCGAGCTGGCCGGCGGCAC) and 6735GER (GTGCCGCCGGCCAGCTCGCAGCCTATGCGG) and the asymmetric overlap extension PCR method for site-directed mutagenesis (53). The gene encoding SCO5461 protein (NCBI, gene identifier 1100901) was amplified using primers 5461F (GCCGCCCATATGCCGTCGGCTGCCCCCGCAAG) and 5461R (GAGCCAGGATCCCCGGTGTCAGTGCCAGGGC) and cloned into pET15b. These primers were designed to skip the first 102 nucleotides (that correspond to the first 34 amino acids spanning the predicted transmembrane region). The resulting plasmid constructs were verified by sequencing and introduced into *E. coli* BL21(DE3). Overexpression of the recombinant SCO6735 gene in the 2-liter culture was induced with 0.1 mm isopropyl β-d-thiogalactopyranoside at *A*_600_ = 0.8 and continued at 16 °C overnight. Overexpression of the recombinant SCO5461 gene in the 500-ml culture was induced with 0.8 mm isopropyl β-d-thiogalactopyranoside at *A*_600_ = 0.8 and continued at 30 °C for the next 3 h. Bacteria were harvested by centrifugation, resuspended in buffer P (25 mm Tris-HCl, pH 7.5, 500 mm NaCl) containing 10 mm imidazole and 1 mg/ml lysozyme and disrupted by sonication (5 × 30 s). After removing cellular debris by centrifugation at 13,000 × *g* for 30 min, His-tagged recombinant proteins were purified by TALON metal affinity chromatography (Clontech). TALON resin was washed two times in buffer P containing 10 and 20 mm imidazole, whereas elution was performed with the same buffer containing 200 mm imidazole. Purified proteins fractions were pooled, desalted on PD10 columns (GE Healthcare), and stored in buffer containing 25 mm Tris-HCl (pH 7.5), 50 mm NaCl, 1 mm EDTA, 1 mm DTT and 10% glycerol (v/v). PARP1 E988Q mono-mutant was purified as described previously (7). Recombinant PARG was purified as described previously (54). pGEX-4T1 GST-PARP10cd (amino acids 818–1025) was purified as previously described (55) with slight modifications. Briefly, after binding of GST-tagged PARP10 on glutathione-Sepharose beads (GE Healthcare), the protein was extensively washed in lysis buffer and equilibrated in PARP10 reaction buffer (50 mm Tris-HCl, pH 7.5, 75 mm KCl, 4 mm MgCl_2_, 0.25 mm DTT). Protein was kept on beads for the automodification reaction.

##### Gene Disruption and Complementation

Gene disruption was done by replacing the entire coding region of SCO6735 gene with the apramycin resistance cassette using the REDIRECT PCR targeting system (34). The SCO6735 disrupting cassette was generated by PCR using plasmid pIJ773 as a template and specific primers 6735F (GGGGCCACTGTCAGTGGTGGCCCGTACGGTGGCGTCATGattccggggatccgtcgacc) and 6735R (ACGAACGACGTGCACGAGCACTGAGCCGCGGACGGCCTAtgtaggctggagctgcttc), which match the sequences adjacent to the SCO6735 coding region ending in start/stop codons (capital letters) and right/left end of the disruption cassette (lowercase letters). PCR product was used to transform *E. coli* strain BW25113/pIJ790 containing *S. coelicolor* cosmid St5F2A (carrying the SCO6735 gene). After recombination, a strain with mutated cosmid was selected, cosmid was isolated, introduced into *E. coli* ET12567/pUZ8002 to avoid methyl-sensing restriction system, and transferred to *S. coelicolor* by conjugation. Exconjugants were screened for double cross-over recombinants and verified by PCR (using primers T6735F: GTGCTGCTGCTGCCCGTG and T6735R: CTGTTCCAGCCGTCGAAG), genomic Southern analysis, and qRT-PCR.

For the complementation analysis SCO6735 gene was amplified by PCR using *S. coelicolor* genomic DNA as a template and specific primers with introduced SpeI and EcoRV restriction sites (6735SpeI: TCGCGCACTAGTCCGGGCAGGAACGGCCGGCGCC and 6735EcoRV: GTGCACGATATCTGAGCCGCGGACGGCCTAGGC) and cloned into the site-specific integrating vector pMS82 carrying *attP-int* locus derived from the phage ϕBT1 (52). Resulting plasmid construct (pMS82-SCO6735) was verified by sequencing, introduced into *S. coelicolor* Δ6735 strain by conjugation, and integrated into the phage ϕBT1 *attB* integration site via double cross-over.

##### Actinorhodin Quantification

Intracellular and extracellular actinorhodin contents were quantified following the protocol (56) with a single modification using 1 m NaOH instead of 1 m KOH. Intracellular actinorhodin content was quantified from 1-ml culture pellet, whereas supernatant was used to quantify the extracellular γ-actinorhodin. Bacteria were lysed with NaOH, and actinorhodin was precipitated with HCl. Actinorhodin pellet was suspended in 1 m NaOH, and *A*_640_ was measured. Concentrations were calculated according to the Lambert-Beer's law using molar extinction coefficient of the pure actinorhodin in NaOH (ϵ_640_ = 25,320 liters mol^−1^ cm^−1^). All quantifications were done on three independent biological replicates of each of the genotypes.

##### RNA Isolation and Genes Expression Quantification by qRT-PCR

The total RNA was isolated from 100-mg culture pellets using the RNeasy mini kit (Qiagen) following the user manual. Genomic DNA was degraded on column using DNase I provided with the same kit. The quality of isolated RNA was examined by agarose gel electrophoresis and spectrophotometrically, whereas the absence of DNA was confirmed by PCR. 1 μg of total RNA isolated from each sample was subjected to reverse transcription using the high capacity cDNA reverse transcription kit (Applied Biosystems). *S. coelicolor* 16S rRNA housekeeping gene was used for normalization (57). The SCO6735 gene was used as a negative control for the SCO6735 knock-out mutant. The following primers were used in qRT-PCR analyses: SCO5085F, GTAATTTCGCATCCGCTGAAC; SCO5085R, GGAGATTCCGATACGATTCCAG; SCO5087F, GAAGGAGCTGTTCGGATTGAAG; SCO5087R, AGGTGAGCAGTTCCCAGAA; 16SF, GCGGCGGAGCATGTGGCTTA; 16SR, CACCTGTACACCGACCACAA; RT6735F, GGCTGGGGCAAGGGCTTCGT; and RT6735R, GCGCCGAGACCGAAGTCGTT.

All primer pairs were tested *a priori*, and their efficiency was measured as described (58) and used for the analyses of mean normalized expression (58). Quantitative real time PCRs were performed in 10-μl volume using SYBR Green PCR Master Mix (Applied Biosystems), 900 nm primers (each), and 1.5 μl of cDNA. The thermal profile consisted of 10-min initial denaturation at 95 °C followed by 40 cycles of 15 s at 95 °C and 1 min at 60 °C. Each RNA sample was quantified two times in independent experiments. Amplifications were done using the ABI PRISM Sequence Analyzer 7300 (Applied Biosystems). Quantification results were examined using SDS7300 software version 1.4 (Applied Biosystems). Statistical analyses were done using *R* version 3.2.1. Mean normalized expression (MNE) of the genes whose deregulation was tested by qRT-PCR was calculated using the formula as described (58),

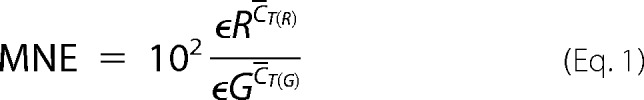
 where ϵ is the efficiency of the primer pair amplifying either the reference (*i.e.* housekeeping) gene (*R*) or the gene tested for the deregulation (*G*), and [overbar]*C*[/overbar]_T_ is the mean value of threshold cycle in qRT-PCR. All quantifications were done on at least three independent biological replicates of each genotype.

##### UV and MMS Treatment

*S. coelicolor* spores suspended in 20% glycerol (v/v) were filtered through 1.2-μm filters (Sartorius) to obtain a single-spore suspension. For testing UV sensitivity, 10 ml of single-spore suspension in 20% glycerol (v/v) was poured into a 9-cm glass Petri dish and irradiated with a constant dose of UV light (50 J m^−2^) six times using Philips 30 W (254 nm) low pressure mercury lamp. The dose was measured by VLX-3 W radiometer. The samples were taken after each doze of irradiation, and the total dose was calculated as the sum of all received doses. For testing MMS sensitivity, raising volumes (from 0.1 to 1% (v/v)) of MMS stock solution (1.3 g/ml; Sigma) were added to the aliquots of spores suspension and incubated at 30 °C for 30 min. The reactions were terminated by diluting the spores 1:10 in 0.16 m sodium thiosulfate (47). After UV irradiation and MMS treatment, serial decimal dilutions of each sample and each dose were spread on tryptic soy broth plates and incubated at 30 °C for 24–48 h. Colony forming units were counted, and survival rates were calculated. For testing deregulation of the SCO6735 gene transcription upon DNA damage, mycelium of *S. coelicolor* wild type strain was irradiated with 200 J m^−2^.

##### Phylogenetic Analysis

Protein sequences were obtained from NCBI non-redundant database using human macrodomain proteins (MacroD1, TARG1, and ALC1) and a bacterial type of PARG as a query (BLAST). Macrodomains were selected from the retrieved protein sequences using SMART database or manually in the case of PARG macrodomains and aligned with the MUSCLE multiple alignment tool, using default settings (59). The multiple alignment was subjected to a maximum likelihood (ML) analysis using MEGA6 (60). The model for ML analysis was selected with ProtTest3 (61) and the Akaike information criterion (62), which indicated the Whelan_And_Goldman model (F + G) (63). Bayesian MCMC analysis was performed in MrBayes version 3.1.2 (64). Bootstrap tests were performed with 1000 replicates. For the structure-based alignment, we used PRALINE (65).

##### Protein Crystallization and Structure Determination

For crystallization, the protein SCO6735 was concentrated up to 10 mg/ml in 25 mm Tris-HCl (pH 7.5), 50 mm NaCl, 1 mm EDTA, 1 mm DTT, and 10% (v/v) glycerol. Initial conditions were identified utilizing sitting drop vapor diffusion method at 18 °C with JCSG III crystallization suite (Qiagen). The best crystals were obtained by hanging drop vapor diffusion method by equilibrating a 1.0-μl drop of protein mixture in a 1:1 ratio with reservoir solution containing 0.1 m Tris-HCl (pH 7.0), 0.29 m NaCl, and 1.0 m sodium citrate. Crystals grew in 5–14 days at 18 °C. Prior to flash cooling the crystals were soaked for few seconds in the reservoir solution containing 20% ethylene glycol as additional cryoprotectant. The crystals contain one macromolecule in the asymmetric unit corresponding to *V*_m_ = 9.85 Å^3^ Da^−1^ and solvent content of 52.25%.

Single crystal x-ray data collection was done on Dectris Pilatus 2M area detector at Elettra Sincrotrone Trieste, Beamline 5.2R (Table 1). Data processing was performed with XDS (66), and data scaling was performed with Aimless (67) within CCP4 (68) software suite. The structure was determined by molecular replacement with program MOLREP (69), using unknown protein from *B. thetaiotaomicron* as a model (PDB code 2FG1) obtained by Phyre2 (70). Initial model was improved by several cycles of refinement, using programs REFMAC (71, 72) and Phenix (73). Data collection and refinement statistics are given in Table 1. Final coordinates and structure factors have been deposited in the Protein Data Bank (accession number 5E3B).

**TABLE 1:** **Data collection and refinement statistics**

	SCO6735
**Data collection**	
Space group	*P* 4_3_2_1_2
Cell dimensions: *a*, *b*, *c* (Å)	103.82, 103.82, 33.25
Resolution (Å)	50-1.60 (1.64-1.60)
*R*_merge_*^a^*	0.120 (1.18)
*R*_meas_*^b^*	0.128 (1.26)
*I*/σ(*I*)	11.5 (1.8)
CC_1/2_	0.996 (0.670)
Completeness (%)	97.76 (89.47)
Redundancy	8.6

**Refinement**	
Resolution (Å)	46.43-1.60
No. of reflections	22860
*R*_work_*^c^*/*R*_free_*^d^*	0.18/0.22 (0.26/0.35)
No. of atoms	
Macromolecule	1167
Water	161
Ligand (1 ethylene glycol and 2 Na^+^)	6
*B*-Factor	
Macromolecule	19.31
Water	31.53
Ligand	22.53
RMSD	
Bond lengths (Å)	0.023
Bond angles	2.252
Ramachandran statistics (%)	
Most favored regions	97
Additional allowed regions	2
Outliers	1

*^a^ R*_merge_ = Σ_hkl_Σ_i_|*I*_i_(*hkl*) − <*I*(*hkl*)>|/Σ_hkl_Σ_i_*I*_i_(*hkl*), where *I*_i_(*hkl*) is the intensity of a given reflection, and <*I*(*hkl*)> is the mean intensity of symmetry-related reflections.

*^b^ R*_meas_ = Σhkl✓*n*/(*n* − 1)Σ_*i*=1_^*n*^|*I*_hkl,i_ − <*I*_hkl_>|/Σ_hkl_Σ_j_*I*_hkl,j_, where *n* is multiplicity. *R*_meas_ is redundancy-independent version of *R*_merge_.

*^c^ R*_work_ = Σ_hkl_||*F*_obs_| − |*F*_calc_||/Σ_hkl_|*F*_obs_|, where *F*_obs_ and *F*_calc_ are the observed and calculated structure factors, respectively.

*^d^ R*_free_ was calculated using 5% of the data set chosen at random that was excluded from the refinement.

##### SCO6735 Activity Assay

To test the hydrolytic activity of SCO6735, de-ADP-ribosylation assay was performed using mono-ADP-ribosylated SCO5461ΔN34 and human PARP1 E988Q mono-mutant (performing only mono-ADP-ribosylation (7)) as substrates. Auto-ADP-ribosylation of SCO5461ΔN34 substrate was performed in the assay buffer (50 mm Tris-HCl, pH 7.5, 50 mm NaCl, 2 mm MgCl_2_) at room temperature using a mixture of cold NAD^+^ (5 μm) and [^32^P]NAD^+^ (2 μCi per reaction) (PerkinElmer Life Sciences). After 30 min, the reactions were split in two, and SCO6735 was added to one aliquot at 5 μm concentration and incubated for additional 30 min. Auto-ADP-ribosylation of PARP1 E988Q mono-mutant was performed as previously described (55, 74), using 0.5 μm of recombinant PARP1 E988Q. After 30 min, reaction were split and treated or not with 1 μm of SCO6735 or SCO6735 G128E mutant. The reactions were stopped by adding SDS-PAGE loading buffer and denatured at 80 °C for 5 min. The reaction products were analyzed by SDS-PAGE and visualized by autoradiography. Quantification of residual radioactive signal on PARP1 E988Q upon treatment with SCO6735 and SCO6735 G128E mutant was performed by ImageJ, normalizing the intensity of autoradiography gel bands for the intensity of corresponding bands visualized on Coomassie staining.

##### Thin Layer Chromatography

To assess the release of [^32^P]ADP-ribose upon treatment with SCO6735, we used recombinant GST-PARP10cd as a substrate. GST-PARP10cd was automodified on beads in PARP10 reaction buffer in the presence of cold NAD^+^ (50 μm) and [^32^P]NAD^+^ (2 μCi/reaction) for 15 min at 37 °C with continuous agitation. After incubation, beads containing GST-PARP10cd were pelleted, washed five times in reaction buffer, and split in two, and SCO6735 was added to one aliquot at 1 μm concentration and incubated for additional 30 min. The beads were pelleted again, and 1 μl of the liquid was phase-loaded on the thin layer chromatography plate (TLC; Macherey-Nagel, Polygram CEL 300 PEI/UV254). As [^32^P]ADP-ribose marker, PARP1 (Trevigen) was automodified, passed three times through G25 columns (GE Healthcare), and treated with recombinant PARG as previously described (55, 74). The plates were developed in 0.15 m LiCl and 0.15 m formic acid. Dried plates were exposed on x-ray film (55, 74).

##### Mass Spectrometry Analysis

Auto-ADP-ribosylation of SCO5461ΔN34 protein (12.5 μm) was performed in the assay buffer (50 mm Tris-HCl, pH 7.5, 50 mm NaCl, 2 mm MgCl_2_) with NAD^+^ (50 μm) at room temperature for 30 min. Proteins were digested with trypsin according to the FASP protocol (75) with a 10-kDa cut-off Vivacon 500 flat ultrafiltration filters (Sartorius Stedim). The resulting peptides were analyzed by UHPLC-MS/MS on an EASY-nLC 1000 liquid chromatography system (Thermo Scientific) coupled online to a Q Exactive HF orbitrap mass spectrometer (Thermo Scientific) essentially as described before (2). The data were processed with MaxQuant (76) using parameters optimized for the detection of ADP-ribosylated peptides (2).

## Author Contributions

J. L. performed actinorhodin quantification and qRT-PCR experiments; M. P. M. purified proteins and performed biochemical analysis; L. P. performed biochemical and TLC analysis; D. P. and H. Ć. performed phylogenetic analysis; I. S. and M. L. performed protein crystallization and x-ray structure determination; R. Ž. performed biochemical analysis; T. C. and I. M. performed MS experiments and data analysis; B. P. performed qRT-PCR experiments; M. H. performed DNA sequencing and sequence analysis; G. J., G. B., and M. A. performed supporting studies; A. M. performed gene cloning, gene inactivation and complementation, and wrote the manuscript; and I. A. designed experiments, analyzed data, and wrote the manuscript.

## Footnotes

4 The abbreviations used are:

PAR: poly-ADP-ribose
PARP: poly-ADP-ribose polymerases
PARG: poly-ADP-ribose glycohydrolase
RMSD: root mean square deviation
ARH: ADP-ribosylhydrolase
ART: ADP-ribosyltransferase
DraG: dinitrogenase reductase-activating glycohydrolase
PDB: Protein Data Bank
qRT-PCR: quantitative real time PCR
ANOVA: analysis of variance
MM: minimal medium
MMS: methyl methanesulfonate
ML: maximum likelihood.
